# Patient and Provider Perspectives on a Novel, Low-Threshold HIV PrEP Program for People Who Inject Drugs Experiencing Homelessness

**DOI:** 10.1007/s11606-022-07672-5

**Published:** 2022-05-25

**Authors:** Angela R. Bazzi, Leah C. Shaw, Katie B. Biello, Seamus Vahey, Jennifer K. Brody

**Affiliations:** 1 Herbert Wertheim School of Public Health, University of California, San Diego, 9500 Gilman Drive, MTF 265E (Mail Code 0725), La Jolla, CA 92161 USA; 2 Department of Community Health Sciences, Boston University School of Public Health, Boston, MA USA; 3 Boston Healthcare for the Homeless Program, Boston, MA USA; 4 Department of Behavioral and Social Sciences, Brown University School of Public Health, Providence, RI USA; 5 Center for Health Promotion and Health Equity, Brown University, Providence, RI USA; 6 Department of Epidemiology, Brown University School of Public Health, Providence, RI USA; 7 The Fenway Institute, Fenway Health, Boston, MA USA; 8 Harvard Medical School, Boston, USA

**Keywords:** HIV infections, homeless persons, substance use, intravenous, pre-exposure prophylaxis, delivery of health care, patient navigation, community health services, harm reduction, public health, program evaluation

## Abstract

**Background:**

HIV outbreaks among people who inject drugs (PWID) and experience homelessness are increasing across the USA. Despite high levels of need, multilevel barriers to accessing antiretroviral pre-exposure prophylaxis (PrEP) for HIV prevention persist for this population. The Boston Health Care for the Homeless Program (BHCHP) initiated a low-threshold, outreach-based program to support engagement in PrEP services among PWID experiencing homelessness.

**Methods:**

To inform dissemination efforts, we explored patient and provider perspectives on key program components. From March to December 2020, we conducted semi-structured qualitative interviews with current and former BHCHP PrEP program participants and prescribers, patient navigators, and outreach workers (i.e., providers). Thematic analysis explored perspectives on key program components.

**Results:**

Participants (*n* = 21) and providers (*n* = 11) identified the following five key components of BHCHP’s PrEP program that they perceived to be particularly helpful for supporting patient engagement in PrEP services: (1) community-driven PrEP education; (2) low-threshold, accessible programming including same-day PrEP prescribing; (3) tailored prescribing supports (e.g., on-site pharmacy, short-term prescriptions, medication storage); (4) intensive outreach and navigation; and (5) trusting, respectful patient-provider relationships.

**Discussion:**

Findings suggest that more patient-centered services formed the basis of BHCHP’s innovative, successful PrEP program. While contextual challenges including competing public health emergencies and homeless encampment “sweeps” necessitate ongoing programmatic adaptations, lessons from BHCHP’s PrEP program can inform PrEP delivery in a range of community-based settings serving this population, including syringe service programs and shelters.

## BACKGROUND

HIV incidence is increasing among people who inject drugs (PWID) in the USA for the first time in decades.^1,2^ With a persistent 7–10% of new HIV infections attributed to injection drug use annually,^2,3^ recent HIV outbreaks among PWID in diverse regions of the country (e.g., Indiana,^4^ Massachusetts,^5,6^ Washington^7^) have been linked to increased opioid and polysubstance use.^8^ Individuals who use and inject fentanyl, now pervasive in illicit drug supplies,^9^ are susceptible to more frequent injection and receptive syringe sharing.^10–12^ Additionally, psychostimulants, which have long been associated with sexual exposure to HIV, may be further contributing to HIV transmission among PWID.^13,14^ New HIV prevention strategies are urgently needed.

Antiretroviral pre-exposure prophylaxis (PrEP) is drastically underutilized among U.S. PWID.^15^ Although a quarter of HIV-negative PWID reported being aware of PrEP by 2018, only 1% had used it.^16^ This low PrEP uptake stands in stark contrast to high levels of need. For example, among 423 PWID in Boston, MA, 92% reported sexual and injection behaviors aligned with clinical PrEP indicators, yet only 2% had used it.^17^ Multilevel barriers to PrEP uptake include limited knowledge,^15,18–23^ underutilization of traditional clinical settings where PrEP is prescribed, and structural vulnerabilities including stigma, poverty, and homelessness.^24–36^ PrEP prescribers are also less willing to prescribe PrEP to PWID than other at-risk groups.^15,23,37,38^ Improved PrEP programming for PWID—and dissemination of strategies that are feasible, acceptable, and effective—is clearly needed.^39^

Only a small number of PrEP programs for PWID experiencing homelessness have been evaluated. During an HIV outbreak among PWID in Glasgow, Scotland, a program involving intensive PrEP outreach and cross-agency collaboration resulted in 32 PWID experiencing homelessness initiating PrEP in 2 years, representing 78% of PrEP-eligible individuals approached in that period.^40^ In Boston, MA, where HIV transmission among PWID experiencing homelessness has increased,^41,42^ the Boston Health Care for the Homeless Program (BHCHP) developed a low-threshold PrEP program that successfully linked 239 individuals to PrEP services in less than 2 years, with a cumulative probability of PrEP persistence at 6 months (assessed via prescription refills) of 44%,^43^ a level similar to those observed in other, more stably housed populations.^44^

While strategies deployed in BHCHP’s PrEP program, including same-day PrEP prescribing and street-based navigation supports, likely contributed to these promising results, from the quantitative assessment described above, little information was available on the acceptability or perceived effectiveness of key program components. We thus conducted a qualitative study to identify key program components that participants and providers perceived as most helpful in supporting PrEP utilization, with the ultimate goal of informing subsequent research and programmatic efforts involving innovative PrEP delivery for PWID experiencing homelessness.

## METHODS

### Program Description

Before October 2018, PrEP delivery at BHCHP involved multiple in-person appointments for laboratory testing, review of results, and provision of 30-day prescriptions.^43^ Follow-up monitoring in primary care was encouraged, but patient navigation and adherence supports were lacking. In fall 2018, a cluster of new HIV infections was detected among local PWID experiencing homelessness, leading to the establishment of a full-time PrEP navigator role to enable flexible PrEP intake and follow-up appointments (including outreach-based laboratory testing), intensive follow-up via phone- and street-based outreach, assistance accessing prescriptions, and supported referrals. Additional strategies implemented by fall 2019 included same-day PrEP initiation, shorter-term prescriptions, and the establishment of “on-demand” access to PrEP via a group of prescribers with PrEP expertise who were available in real-time via secure text messaging. BHCHP also instituted medication storage within a service providing medical monitoring for over-sedation from substance use (the “Supportive Place for Observation and Treatment,” SPOT)^45,46^ and the Boston Public Health Commission’s “engagement center,” a drop-in space offering basic amenities and clinical services.^47^ Aligned with “low-threshold,” harm-reduction-oriented approaches, missed appointments did not trigger medication discontinuation if appropriate laboratory follow-up could be performed. By fall 2020, amidst COVID-19 and ongoing HIV transmission, BHCHP increased telehealth, active HIV testing, and case finding, and nurse-facilitated daily medication dosing in outreach (e.g., engagement center) settings.

### Study Design and Sample

Informed by the principles of grounded theory,^48^ from March to December 2020, we conducted qualitative interviews with BHCHP PrEP patients (hereafter, “participants”) and prescribers (e.g., physicians, nurses), patient navigators, and outreach workers (hereafter, “providers”). Participants were eligible if they were ≥ 18 years old, currently experiencing homelessness, and current or former patients of BHCHP’s PrEP program, and had injected drugs in the past month. We purposively sampled participants for diversity in socio-demographics (e.g., age, race/ethnicity, gender) and PrEP experiences (e.g., duration of use).^49^ BHCHP personnel briefly explained the study goals and methods during routine in-person service encounters and then provided introductions to research staff who were present via secure video-conferencing in private areas of BHCHP. Research staff then conducted eligibility screening and verbal informed consent. We sampled providers for diversity in program role (e.g., clinicians [physicians, nurses], navigators, other program staff). Providers were recruited via email and provided verbal informed consent. Participants received $25; providers were not compensated. The Boston University Medical Campus institutional review board reviewed and approved all study protocols.

### Data Collection

Four research staff with graduate-level training, supervised by a lead qualitative investigator, administered brief quantitative surveys. Surveys assessed participants’ socio-demographics, past-month substance use and sexual behaviors, and service utilization (Table 1) and providers’ professional roles and years of experience working with PWID and in HIV services (Table 2). Research staff then conducted one-time qualitative interviews using semi-structured interview guides informed by previous studies, literature, and community collaborators’ input.^23,24^ Open-ended questions with detailed probes explored the following key domains: drug use behaviors, health concerns, HIV–related risk perceptions, healthcare utilization, and perspectives on and experiences using PrEP and related BHCHP services. On average, interviews lasted 30 min (range: 15–49) with participants and 45 min (range: 25–71) with providers. Interviews were audio-recorded and professionally transcribed. Staff wrote detailed field notes immediately after interviews using a structured template. From weekly meetings and review of interviewers’ notes, we ceased recruitment when deciding as a team that additional data collection would not yield substantially new findings.^50^

**Table 1:** Characteristics of BHCHP PrEP Program Participants Who Inject Drugs and Are Experiencing Homelessness (*n* = 21)

*Age in years, median (interquartile range; IQR)*	36 (31–38)
*Hispanic or Latino*	4 (19%)
*Racial identity*	
American Indian or Alaska Native	1 (5%)
Black or African American	3 (14%)
Other	4 (19%)
White	13 (62%)
*Gender identity*	
Female	6 (29%)
Male	15 (71%)
*Housing, most of the time, past month*	
Street	14 (67%)
Shelter	5 (24%)
Other (e.g., motel, supportive housing)	2 (10%)
*Sexual orientation*	
Heterosexual	18 (86%)
Bisexual	2 (10%)
Homosexual or gay	1 (5%)
*Currently taking PrEP*	16 (76%)
Median duration currently using PrEP, in weeks (n = 16 currently taking PrEP; IQR*)	6 (1–33)
*Median number of sexual partners, past month (n = 15 sexually active participants; IQR)*	1 (2–6)
*Engaged in sex work, past month*	7 (33%)
*Frequency of condom use with sexual partners, past month (n = 15 sexually active participants)*	
Sometimes/rarely/never	11 (73%)
Often/always	4 (27%)
*Drugs used, past month*	
Heroin and/or fentanyl	21 (100%)
Cocaine	19 (90%)
Crack	18 (86%)
Crystal methamphetamine	20 (95%)
Benzodiazepines (e.g., Valium, Ativan, Xanax, Klonopin)	17 (81%)
Marijuana	16 (76%)
Alcohol	8 (38%)
Gabapentin (“Johnnies”)	12 (57%)
Synthetic cannabinoids (“K2,” “spice”)	5 (24%)
“Street” methadone or buprenorphine (not prescribed to you)	7 (33%)
Prochlorperazine (“phenergin”) or clonidine	5 (24%)
Other drugs (e.g., prescription opioids/painkillers, “ecstasy”/MDMA)	4 (19%)
*Frequency of injecting drugs, past month (n = 20 with complete data)*	
10 or more times a day	4 (19%)
7 to 9 times a day	7 (33%)
4 to 6 times a day	6 (29%)
2 to 3 times a day	1 (5%)
One daily or less	3 (14%)
*Drugs injected, past month (n = 20 with complete data)*	
Heroin and/or fentanyl	20 (95%)
Cocaine	15 (71%)
Crack	11 (52%)
Crystal methamphetamine	16 (76%)
Benzodiazepines (e.g., Valium, Ativan, Xanax, Klonopin)	2 (10%)
“Street” methadone or buprenorphine (not prescribed to you)	3 (14%)
*Distributive syringe sharing, past month*	
Sometimes/rarely/never	12 (57%)
Often/always	9 (43%)
*Receptive syringe sharing, past month*	
Sometimes/rarely/never	16 (76%)
Often/always	5 (24%)
*Sharing of other injection equipment (e.g., cookers, cottons, rinse water), past month*	
Sometimes/rarely/never	5 (24%)
Often/always	16 (76%)
*Sources of sterile syringes, past month*	
Syringe exchange (SSP)	19 (90%)
Other people	5 (24%)
Other (e.g., homeless engagement center, health center, pharmacy)	9 (43%)
*Ever been told by a doctor that you have hepatitis C virus (HCV)*	20 (95%)
*Median number of years since a doctor told you that you have HCV (n = 20 ever told about having HCV; IQR)*	10 (5–12)

**Table 2:** Characteristics of BHCHP PrEP Program Providers (*n* = 11)

*Job types (not mutually exclusive)*	*n (%)*
Clinical roles (e.g., physician, physician’s assistant, nurse practitioner, registered nurse)	7 (64%)
Non-clinical roles (e.g., case manager, program coordinator, outreach worker)	5 (45%)
*Median years working (interquartile range; IQR)*	
At organization	4 (3–6)
Professionally with PWID	6 (5–13)
In HIV prevention/treatment	6 (3–11)
With PrEP services	2 (2–4)

### Data Analysis

We collaboratively developed a codebook by independently reading three selected transcripts to generate deductive and inductive codes with definitions, which were then discussed and refined as a team.^51,52^ We independently tested preliminary codes on two different transcripts and subsequently met to revise the codebook. Through three additional rounds of this process, we reached consensus on a final codebook. Then, one analyst applied final codes to all transcripts using NVivo (v12) and met weekly with the team to discuss coding progress and potential themes. In-depth, thematic analysis for this paper then involved synthesizing codes for PrEP knowledge, uptake, adherence, and retention in care to identify key program components that participants and providers perceived as supporting PrEP utilization. We illustrate key findings using representative quotes and pseudonyms.

## RESULTS

### Participant Characteristics

Among 21 PrEP program participants, median age was 36 years (interquartile range [IQR]: 31–38), 15 (71%) identified as male, 16 (76%) were currently taking PrEP, and median duration currently taking PrEP was 6 weeks (IQR: 1–33; Table 1). Drugs commonly used in the past month included heroin and/or fentanyl (100%), methamphetamine (95%), cocaine (90%), crack (86%), and non-prescribed benzodiazepines (81%). Injection frequency was high, with half (52%) injecting at least seven times daily and 19% injecting 10 or more times daily. Most (76%) reported sharing injection equipment in the past month. Among 11 providers, median years of experience working with PWID was six (IQR: 4.5–13; Table 2). Providers’ roles were clinical (medical doctor, physician’s assistant, nurse practitioner, and registered nurse) and non-clinical (program coordinator, case manager, and outreach worker).

### Perspectives on Key Program Components

From semi-structured interviews, we identified five key components of BHCHP’s PrEP program that participants and providers perceived to be particularly helpful for supporting patient engagement in PrEP services: (1) community-driven PrEP education; (2) low-threshold, accessible PrEP programming; (3) additional PrEP prescribing supports; (4) intensive outreach and navigation; and (5) trusting patient-provider relationships.

#### Community-Driven PrEP Education

Participants and providers described how BHCHP’s PrEP education facilitated patients’ connections to PrEP services. Importantly, education was provided by trusted sources including BHCHP’s PrEP navigator, who Matthew, a 34-year-old man, described as consistently supportive: *“He’s the one that brought [PrEP] up to me initially and he’s never switched it up; he’s always the same cool dude, trying to help me out.”* Other participants like David, a 39-year-old man, learned about PrEP via *“word of mouth”* from trusted peers who were PrEP patients: *“[We] like learning from one addict to another, not from staff, so we can relate better. So hearing from people who are on [PrEP] is really important.”* A non-clinical provider reiterated that peer-delivered PrEP education was *“the best way to disseminate [information] in this community…it’s something that we [providers] don’t have a say [in] because we’re not in that community.”*

**Fig. 1:**
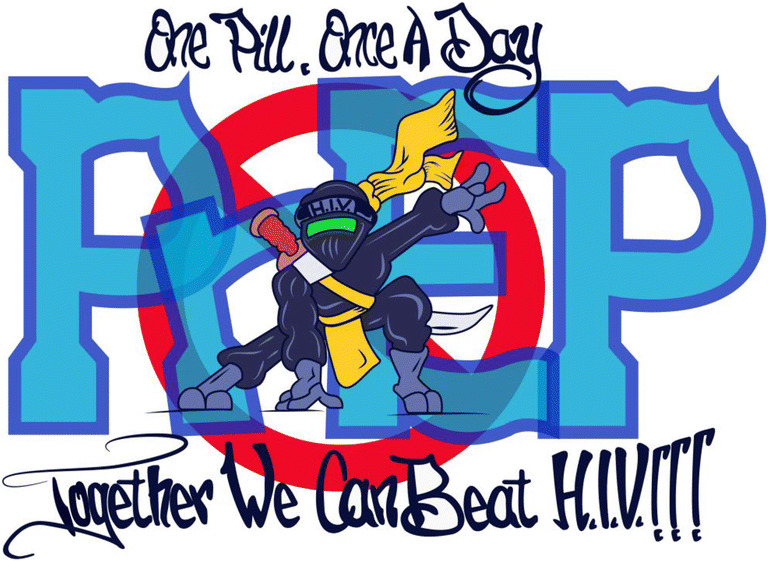
PrEP marketing image developed by BHCHP PrEP program participants and a collaborating syringe service program. Credit: Nava Shaw.

With direct community input, educational materials were tailored for patients’ needs, resulting in materials that were more relevant and influential than those developed by health departments and industry. Brandon, a 34-year-old man, explained, *“I thought [PrEP] was only for gay guys [and] sexual activity, and that threw me off and made me not interested in it.”* Michael, a 44-year-old man, described how BHCHP and collaborating syringe service program (SSP) personnel engaged him in the development of materials (see Fig. 1):

*There’s a lot of misinformation about PrEP from earlier marketing, and stigma that it’s for people with HIV…To design different materials, we had an artist and little catch sayings…so we were a part of designing new stuff, and I’ve seen it around.*

A clinician also emphasized how messaging addressed *“urban legends,”* kept it *“simple,”* aligned with the overall harm-reduction orientation of the program: *“We want to figure out where they’re at, what they understand, and what the gaps are.”*

Widespread community dissemination of PrEP information, whether in print or via word of mouth through collaborating SSPs, homeless shelters, tent encampments, drug detoxification centers, and methadone clinics, was also helpful. Matthew described how BHCHP personnel *“came out to the tents with information about PrEP, how easy it is to use, and all the reasons why it would be beneficial.”* Clinical providers described creative ways to disseminate PrEP information by leveraging routine service encounters and trusted reputations:

*[We] talk about PrEP all the time...We made necklaces out of unused PrEP [pills] to engage people’s interest. And when I go out to the corner, I’m like, “Hey, I’ve got socks! By the way, there’s been 90 new [HIV] cases here recently so you need to know about PrEP.” It’s that way of engagement. [And] they know me.*

#### Low-Threshold, Accessible PrEP Programming

Participants and providers described two ways that BHCHP’s low-threshold programming increased PrEP access. First, BHCHP integrated PrEP referral systems and personnel into a range of existing services that patients routinely accessed, including primary care, wound care, case management, behavioral health, treatment for opioid use disorder, and medical monitoring for over-sedation (i.e., the “SPOT”). A clinician described this approach:

*To offer [PrEP] to more patients, we bring it up frequently, whenever the subject is injecting drugs…First, I ask permission, obviously, like, “Is it OK to talk with you about HIV prevention?” Then, if so, “When was the last time you got checked for HIV?” That’s a low barrier question that people like, and it’s an entrée into the PrEP discussion.*

Participants also described being referred to PrEP services while *“getting blood work done”* through street-based outreach or from the addiction treatment nurse *“who sees me daily and knows about my lifestyle,”* representing comfortable, familiar environments. Another clinician emphasized the significance of street-based PrEP screening: *“More providers need to understand how beneficial it is coming to where people are, and recognize the barriers [for] people coming into the clinic. We have to be more understanding that that is really very difficult.”*

Second, same-day PrEP prescribing facilitated patients’ PrEP uptake. A clinical provider explained how, as HIV transmission among local PWID increased over time, the PrEP program *“become even more low barrier”* and *“seized the opportunity”* to reach additional patients:

*Initially we had “PrEP champions” assigned to anybody injecting drugs who walked in and wanted PrEP…Then we started [HIV] testing in shelters, and had street-based conference calls, essentially an on-the-spot telephonic [PrEP] start, even before we had the lab results. So that day, we would write a seven-day PrEP script, and then follow up in a week…that definitely increased our PrEP starts.*

Patients appreciated the ease of this same-day PrEP prescribing, which David described as *“very easy; they gave me medication the same day I asked for it.”*

#### Additional PrEP Prescribing Supports

Another key program component involved BHCHP’s supports for accessing PrEP prescriptions. Recognizing the difficulty of safeguarding medications for patients experiencing homelessness, BHCHP’s on-site pharmacy repackaged PrEP into 7-day supplies, which Michael described as supporting his adherence and making PrEP use less stressful: *“I basically live out of a backpack [without] safe places to keep a 30-day script…If my backpack comes up missing, they have another 21 days for me. It prevents that lapse, promotes my continually taking it, and gives me a little breathing room.”* A clinician emphasized how these prescribing supports required staff time and dedication:

*It’s commitment [by] pharmacy staff. To get approval every time, [you’re] sitting on the phone with the insurance company…That’s a lot of time. Your average [commercial pharmacy] isn’t going to do that, but the pharmacy here is really accommodating for our patients.*

In contrast to experiences in other pharmacies, in which participants described as uncomfortable, stigmatizing, and as though *“everybody’s watching me,”* interactions with BHCHP’s pharmacists were described positively. Jennifer, a 42-year-old woman, recognized the staff commitment when her prescription was stolen: *“You have no idea, the dedication…they come through and get [a replacement] for me again; they’re awesome.”*

On-site medication storage, which was available through SPOT and the engagement center, was another important support for patients’ PrEP access and adherence, as Michael explained: *“I’m visiting [BHCHP] all the time. They’ll ask me, ‘Have you taken your medication today?’ And I’ll say, ‘Shit, you know what? I haven’t, but thanks for reminding me,’ and I’ll go get it right now.”* Participants also described how BHCHP’s medication storage supported their PrEP adherence, saying, *“It’s about the only thing that helps,” “If it wasn’t for the [PrEP storage], I wouldn’t be taking it daily,”* and *“When we can leave our meds here, there’s really no excuse to not take it daily.”* A clinician also explained how on-site medication storage was critical for PrEP access and adherence:

*We always ask [patients] if they’re concerned about losing their meds. That answer’s always yes…For starting PrEP, that’s one of our first questions. Then, “Where do you want us to hold onto it? Where are you most likely going to see us every day?” As long as we have that conversation, generally, people leave PrEP with us.*

#### Intensive Outreach and Navigation

Intensive street-based outreach by BHCHP personnel also supported patients’ PrEP adherence and persistence, as Crystal, a 36-year-old woman, explained: *“[The PrEP navigator] knows where to find me and reminds me of appointments and taking [PrEP]…I would forget if I didn’t have someone behind me, and I wouldn’t care; I wouldn’t do any of it.”* Consistent outreach also supported some participants like Matthew with re-initiating PrEP: *“I got offered [PrEP] before, took it the first day, but then never went back. But this time I’ve been following through and they’ve been making it really easy for me because [the navigator] is always around, doing everything he can to try to help us.”* Clinical providers also emphasized the importance of PrEP navigator support, especially for patients who were less engaged in clinical services:

*As a primary care doctor, a concern starting someone on PrEP is like, who is going to [get] their labs and track them? It’s a little nerve-racking, wondering who’s followed up, how many are still taking PrEP, who’s due for labs? So it’s nice to know that someone is keeping track and calling people if they’re overdue…It’s a nice backup system.*

An extension of BHCHP’s intensive PrEP outreach was daily medication dosing. Slightly distinct from traditional models of directly observed therapy, BHCHP’s approach involved nursing staff delivering single PrEP doses to patients, typically with a juice box, and often (but not always) observing participants take their dose. A clinician explained the utility of this approach because *“patients are not used to coming up to us,”* and due to the competing priorities of homelessness and addiction, many participants like Diego, a 38-year-old man, appreciated this support, saying, *“I’ve been taking [PrEP] without fail because every time they see me, they give it to me, and I take it.”* A clinician emphasized the importance of approaching people with respect within their daily medication dosing strategy:

*For the folks who just absolutely can’t take [PrEP] daily, they know they can’t, and they’re like, “You take my meds’ you make sure I take them.” Those are the people we’re searching out on the street...The nurses will literally deliver one pill for today, and then again tomorrow…We get people’s agreement first, like, “Is it OK if I walk up to you and ask you about your pills?” And they’ll say, “Yeah, sure.”*

#### Trusting Patient-Provider Relationships

A final program component that emerged from interviews involved the cultivation of trusting, respectful patient-provider relationships. Jennifer viewed these positive relationships as supporting her PrEP persistence, saying, *“It goes way beyond the medication. It’s the people that are involved…They’re personable, really comfortable to be around, and [they] have never changed who they were, not once. And that’s a big part of why I’m still so involved with [PrEP].”* Several providers, including the following clinician, also described the importance of trusting relationships and the need to create a *“safe space”* for private discussions, especially for men engaged in sex work:

*We see [our patients] every day. They have significant distrust in the medical establishment, but they know us and trust us…So eventually, if we’ve established enough trust, it’s easier to talk about uncomfortable [or] very private things, like [sex work]…So it’s just constant engagement and creating a safe space. And [they] know everything’s confidential; we’re not sitting there typing everything into their [medical] record. It’s just a safe space for everyone.*

A non-clinical provider also explained that building trusting relationships required being *“proactive”* and *“going out to them to engage them in conversation, respecting who they are, and saying, ‘You deserve better than this,’ which I don’t think is a common phrase in these populations.”* Matthew echoed the significance of providers’ efforts, saying, *“In this lifestyle, being personable goes a long way. When you’re homeless and getting high, you don’t trust anybody; you don’t have anybody. So when somebody’s taking time out of their day to try to help you, you take it seriously.”*

## DISCUSSION

In the context of ongoing HIV transmission among PWID experiencing homelessness across the USA,^2^ programmatic innovations are needed to expand access to effective HIV prevention options for this population.^39,53^ Most PrEP research with PWID has been with individuals lacking PrEP experience, and beyond research contexts,^54^ very few real-world programs have been investigated.^40,43^ From interviews with PrEP patients and providers involved with a novel program for PWID experiencing homelessness in Boston, MA, we identified key program components that were perceived to be acceptable, feasible, and supportive of patients’ success. While some aspects of this program are unique, our findings could be informative for implementation research and practice in other settings such as community health centers, SSPs, and shelters.

First, we found that BHCHP’s facilitation of community-driven PrEP education appeared more relevant and influential than existing materials developed by health departments and industry, which participants viewed as overly focused on sexual risk, limiting their PrEP interest.^23^ With collaborating agencies, BHCHP engaged patients in developing educational materials tailored for their patient population (Fig. 1). BHCHP also deployed trusted providers to deliver this education within clinical services and street-based outreach. Finally, PrEP education delivered by peers (i.e., via “word of mouth”) was motivational for participants, suggesting that research should further explore the potential for peer-delivered PrEP education and referrals throughout patients’ social networks.^55–57^

Next, BHCHP’s accessible PrEP programming facilitated initial PrEP uptake. The integration of PrEP-related conversations and referrals into routine service encounters within comfortable, familiar environments (e.g., the “SPOT”) connected patients to PrEP, standing in stark contrast to PrEP services requiring patients to navigate health systems to reach prescribers. BHCHP’s low-threshold PrEP programming required taking PrEP services, including laboratory testing, into the community, rather than expecting patients to access clinical settings. This shift may be highly significant for patients experiencing stigma and distrusting medical institutions,^25^ and similar services could be implemented in other community settings (e.g., SSPs, shelters). BHCHP’s same-day PrEP prescribing protocols also reduced or eliminated requirements for patients to make and attend multiple appointments before initiating PrEP. Reducing barriers to PrEP uptake is clearly important for this population, as only 2% of PWID in the Greater Boston Area^17^ and < 1% nationally have ever accessed PrEP.^16^ These protocols also reflected providers’ awareness of the need to “seize the opportunity” to discuss and prescribe PrEP whenever patient encounters occurred, rather than relying on appointment-based systems.^10^

Additional prescribing supports tailored for the needs of PWID experiencing homelessness supported patients’ ongoing PrEP adherence and persistence. These supports included an on-site pharmacy, short-term PrEP prescriptions, assistance with medication replacement, and medication storage in convenient, accessible locations. While pharmacies can support HIV prevention initiatives,^58^ our findings suggest that non-stigmatizing, easily accessible prescribing supports are needed to help individuals in this population with PrEP adherence. Similar to lessons learned from an evaluation of PrEP services for PWID in Glasgow, which utilized specialty staffing and facilities already serving the target population, leadership and financial supports will be critical to the implementation and sustainability of these prescribing supports.^40^

BHCHP’s intensive, street-based outreach and navigation (with daily medication dosing) became critically important in the context of repeated street “sweeps” of homeless encampments throughout Boston. These operations are increasingly common nationally and typically involve police or government agencies forcibly moving people experiencing homelessness,^59,60^ often confiscating or destroying personal belongings including medications.^61,62^ BHCHP’s provision of medication storage and daily dosing, while labor intensive, likely helped patients avoid medication loss and theft. Although long-acting injectable PrEP could reduce the need for this level of daily outreach, frequent contact with patients can also help retain patients in care, enable referrals to other services, and support the trusting patient-provider relationships that are critical for this often-stigmatized population.^25^

Limitations of our study include our focus on a single program within a unique organization and external context, possibly reducing generalizability. As Massachusetts has high insurance coverage and generally strong support for public health initiatives, future studies, ideally with a broader range of informants, should investigate implementation barriers and facilitators in settings where internal and external supports may differ. Our recruitment approach through BHCHP may have also resulted in a sample with largely positive perspectives. Although we included individuals disengaged from services and our qualitative approach was open to negative feedback, we were unable to identify substantial unmet needs, ongoing barriers, or program-related reasons for PrEP discontinuation. Finally, we did not formally verify patient outcomes using review of medical records or PrEP navigator notes; larger quantitative studies involving these data sources could help connect specific program components to patient outcomes.

Despite these limitations, this study was the first to our knowledge to investigate how low-threshold PrEP programming innovations can support successful and sustained engagement in PrEP services among PWID experiencing homelessness. Additional research and funding resources at the local, state, and national levels will be needed to support eventual scale-up of BHCHP’s innovative strategies. While contextual challenges including competing public health emergencies and forcible relocation of people experiencing homelessness are requiring ongoing programmatic adaptations, specific aspects of BHCHP’s PrEP program could be considered within a range of community settings (e.g., health centers, SSPs, shelters) and for improving services for other health conditions (e.g., hepatitis C virus) that are common in this population.

## References

[1] Broz D, Carnes N, Chapin-Bardales J (2021). Syringe services programs' role in ending the HIV epidemic in the U.S.: why we cannot do it without them. Am J Prev Med.

[2] Handanagic S, Finlayson T, Burnett JC, Broz D, Wejnert C (2021). National HIVBSSG. HIV infection and HIV-associated behaviors among persons who inject drugs - 23 metropolitan statistical areas, United States, 2018. MMWR Morb Mortal Wkly Rep.

[3] Centers for Disease Control and Prevention. Estimated HIV incidence in the United States, 2007–2010. HIV Surveillance Supplemental Report 2012;17(4). Available at: https://www.cdc.gov/hiv/pdf/library/reports/surveillance/cdc-hiv-surveillance-supplemental-report-vol-17-4.pdf. Accessed May 2, 2022.

[4] Gonsalves GS, Crawford FW (2018). Dynamics of the HIV outbreak and response in Scott County, IN, USA, 2011-15: a modelling study. Lancet HIV.

[5] Cranston K, Alpren C, John B (2019). Notes from the field: HIV diagnoses among persons who inject drugs—Northeastern Massachusetts, 2015–2018. MMWR Morb Mortal Wkly Rep.

[6] Alpren C, Dawson EL, John B (2020). Opioid use fueling HIV transmission in an urban setting: an outbreak of HIV infection among people who inject drugs-Massachusetts, 2015-2018. Am J Public Health.

[7] Golden MR, Lechtenberg R, Glick SN (2019). Outbreak of human immunodeficiency virus infection among heterosexual persons who are living homeless and inject drugs—Seattle, Washington, 2018. MMWR Morb Mortal Wkly Rep.

[8] Fauci AS, Redfield RR, Sigounas G, Weahkee MD, Giroir BP (2019). Ending the HIV epidemic: a plan for the United States. JAMA.

[9] Massachusetts Department of Public Health. Data brief: Opioid-related overdose deaths among Massachusetts residents, 2019. Available at: https://www.google.com/url?sa=t&rct=j&q=&esrc=s&source=web&cd=&ved=2ahUKEwiNrqnc7sH3AhVSDkQIHZRkD3QQFnoECAIQAQ&url=https%3A%2F%2Fwww.mass.gov%2Fdoc%2Fopioid-related-overdose-deaths-among-ma-residents-november-2020%2Fdownload&usg=AOvVaw06eSDWCwOZN2ggL93hQw7u. Accessed May 2, 2022.

[10] Taylor JL, Walley AY, Bazzi AR (2019). Stuck in the window with you: HIV exposure prophylaxis in the highest risk people who inject drugs. Subst Abus.

[11] Gladden RM, O’Donnell J, Mattson CL, Seth P (2019). Changes in opioid-involved overdose deaths by opioid type and presence of benzodiazepines, cocaine, and methamphetamine—25 states, July–December 2017 to January–June 2018. MMWR Morb Mortal Wkly Rep.

[12] Lambdin BH, Bluthenthal RN, Zibbell JE, Wenger L, Simpson K, Kral AH (2019). Associations between perceived illicit fentanyl use and infectious disease risks among people who inject drugs. Int J Drug Policy.

[13] Halkitis PN, Mukherjee PP, Palamar JJ (2009). Longitudinal modeling of methamphetamine use and sexual risk behaviors in gay and bisexual men. AIDS Behav.

[14] Brener L, Caruana T, Broady T (2022). Addressing injecting related risks among people who inject both opioids and stimulants: findings from an Australian survey of people who inject drugs. Addict Behav Rep.

[15] Baral SD, Stromdahl S, Beyrer C (2012). The potential uses of preexposure prophylaxis for HIV prevention among people who inject drugs. Curr Opin Hiv Aids.

[16] Centers for Disease Control and Prevention. HIV infection risk, prevention, and testing behaviors among persons who inject drugs—National HIV Behavioral Surveillance: injection drug use, 23 U.S. cities, 2018. HIV Surveillance Special Report 24. http://www.cdc.gov/hiv/library/reports/hiv-surveillance.html. Accessed May 2, 2022, 2020.

[17] Earlywine JJ, Bazzi AR, Biello KB, Klevens RM (2021). High prevalence of indications for pre-exposure prophylaxis among people who inject drugs in Boston, Massachusetts. Am J Prev Med.

[18] Escudero DJ, Kerr T, Wood E (2015). Acceptability of HIV Pre-exposure prophylaxis (PREP) among people who inject drugs (PWID) in a Canadian setting. AIDS Behav.

[19] Escudero DJ, Lurie MN, Kerr T, Howe CJ, Marshall BD (2014). HIV pre-exposure prophylaxis for people who inject drugs: a review of current results and an agenda for future research. J Int AIDS Soc.

[20] Eisingerich AB, Wheelock A, Gomez GB, Garnett GP, Dybul MR, Piot PK (2012). Attitudes and acceptance of oral and parenteral HIV preexposure prophylaxis among potential user groups: a multinational study. Plos One.

[21] Kuo I, Olsen H, Patrick R, et al. Willingness to use HIV pre-exposure prophylaxis among community-recruited, older people who inject drugs in Washington, DC. Drug Alcohol Depend 2016 10.1016/j.drugalcdep.2016.02.044 27177804

[22] Adams ML, Wejnert C, Finlayson T, Xia M, Paz-Bailey G. HIV Infection, risk, prevention, and testing behaviors among persons who inject drugs: National HIV Behavioral Surveillance: injection drug use, 20 US cities, 2015. 2017

[23] Bazzi AR, Biancarelli DL, Childs E (2018). Limited knowledge and mixed interest in pre-exposure prophylaxis for HIV prevention among people who inject drugs. AIDS Patient Care STDS.

[24] Biello KB, Bazzi AR, Mimiaga MJ (2018). Perspectives on HIV pre-exposure prophylaxis (PrEP) utilization and related intervention needs among people who inject drugs. Harm Reduct J.

[25] Biancarelli DL, Biello KB, Childs E (2019). Strategies used by people who inject drugs to avoid stigma in healthcare settings. Drug Alcohol Depend.

[26] Degenhardt L, Mathers B, Vickerman P, Rhodes T, Latkin C, Hickman M (2010). Prevention of HIV infection for people who inject drugs: why individual, structural, and combination approaches are needed. Lancet.

[27] Gonzalez A, Barinas J, O'Cleirigh C (2011). Substance use: impact on adherence and HIV medical treatment. Curr HIV/AIDS Rep.

[28] Malta M, Magnanini MM, Strathdee SA, Bastos FI (2010). Adherence to antiretroviral therapy among HIV-infected drug users: a meta-analysis. AIDS Behav.

[29] Bruce RD (2013). Is it time for treatment as prevention among people who inject drugs?. J Acquir Immune Defic Syndr.

[30] Gonzalez A, Mimiaga MJ, Israel J, Bedoya CA, Safren SA (2013). Substance use predictors of poor medication adherence: the role of substance use coping among HIV-infected patients in opioid dependence treatment. AIDS and Behavior.

[31] Azar MM, Springer SA, Meyer JP, Altice FL (2010). A systematic review of the impact of alcohol use disorders on HIV treatment outcomes, adherence to antiretroviral therapy and health care utilization. Drug and alcohol dependence.

[32] Gonzalez JS, Penedo FJ, Llabre MM (2007). Physical symptoms, beliefs about medications, negative mood, and long-term HIV medication adherence. Ann Behav Med.

[33] Springer SA, Dushaj A, Azar MM (2012). The impact of DSM-IV mental disorders on adherence to combination antiretroviral therapy among adult persons living with HIV/AIDS: a systematic review. AIDS Behav.

[34] Gonzalez A, Barinas J, O’Cleirigh C (2011). Substance use: impact on adherence and HIV medical treatment. Current HIV/AIDS Reports.

[35] Milloy M-J, Montaner J, Wood E (2012). Barriers to HIV treatment among people who use injection drugs: implications for ‘treatment as prevention’. Curr Opin Hiv Aids.

[36] Wood E, Kerr T, Tyndall MW, Montaner JS (2008). A review of barriers and facilitators of HIV treatment among injection drug users. Aids.

[37] Karris MY, Beekmann SE, Mehta SR, Anderson CM, Polgreen PM (2014). Are we prepped for preexposure prophylaxis (PrEP)? Provider opinions on the real-world use of PrEP in the United States and Canada. Clin Infect Dis.

[38] Edelman EJ, Moore BA, Calabrese SK (2017). Primary care physicians' willingness to prescribe HIV pre-exposure prophylaxis for people who inject drugs. AIDS Behav.

[39] Biello KB, Mimiaga MJ, Valente PK, Saxena N, Bazzi AR (2021). The past, present, and future of PrEP implementation among people who use drugs. Curr HIV/AIDS Rep.

[40] Grimshaw C, Boyd L, Smith M, Estcourt CS, Metcalfe R (2021). Evaluation of an inner city HIV pre-exposure prophylaxis service tailored to the needs of people who inject drugs. HIV Med.

[41] Taylor JL, Ruiz-Mercado G, Sperring H, Bazzi AR (2021). A collision of crises: addressing an HIV outbreak among people who inject drugs in the midst of COVID-19. J Subst Abuse Treat.

[42] Massachusetts Department of Public Health & the Boston Public Health Commission. Increase in newly diagnosed HIV infections among persons who inject drugs in Boston, 2019. Available at: https://www.bphc.org/whatwedo/infectious-diseases/Documents/Joint_HIV_in_PWID_advisory_012519%20(1).pdf. Accessed May 2, 2022.

[43] Biello KB, Bazzi AR, Vahey S, Harris M, Shaw L, Brody J. Delivering preexposure prophylaxis to people who use drugs and experience homelessness, Boston, MA, 2018-2020. Am J Public Health 2021;111(6):1045-48. 10.2105/AJPH.2021.306208 [published Online First: Epub Date]|. 10.2105/AJPH.2021.306208 PMC8101577 33950728

[44] Hojilla JC, Vlahov D, Crouch PC, Dawson-Rose C, Freeborn K, Carrico A (2018). HIV pre-exposure prophylaxis (PrEP) uptake and retention among men who have sex with men in a community-based sexual health clinic. AIDS Behav.

[45] Leon C, Cardoso LJP, Johnston S, Mackin S, Bock B, Gaeta JM (2018). Changes in public order after the opening of an overdose monitoring facility for people who inject drugs. Int J Drug Policy.

[46] Wishik G, Gaeta JM, Racine MW, O'Connell JJ, Baggett TP (2021). Substance consumption and intoxication patterns in a medically supervised overdose prevention program for people experiencing homelessness. Subst Abus.

[47] Boston Public Health Commission. Engagement Center. Secondary Engagement Center 2021. Available at: https://www.bphc.org/whatwedo/Recovery-Services/roadmap-to-recovery/Programs-and-Services/Pages/Engagement-Center.aspx. Accessed May 2, 2022.

[48] Corbin JM, Strauss AL (2008). Basics of Qualitative Research. Techniques and Procedures for Developing Grounded Theory.

[49] Johnson JC (1990). Selecting Ethnographic Informants.

[50] Guest G, Bunce A, Johnson L (2016). How many interviews are enough?. Field Methods.

[51] DeCuir-Gunby JT, Marshall PL, McCulloch AW (2010). Developing and using a codebook for the analysis of interview data: an example from a professional development research project. Field Methods.

[52] MacQueen KM, McLellan E, Kay K, Milstein B (2016). Codebook development for team-based qualitative analysis. CAM Journal.

[53] Brody JK, Taylor J, Biello K, Bazzi AR (2021). Towards equity for people who inject drugs in HIV prevention drug trials. Int J Drug Policy.

[54] Roth AM, Tran NK, Felsher M (2021). Integrating HIV preexposure prophylaxis with community-based syringe services for women who inject drugs: results from the project SHE demonstration study. Journal of acquired immune deficiency syndromes.

[55] Rousseau E, Julies RF, Madubela N, Kassim S (2021). Novel platforms for biomedical HIV prevention delivery to key populations - community mobile clinics, peer-supported, pharmacy-led PrEP delivery, and the use of telemedicine. Curr HIV/AIDS Rep.

[56] Walsh T, Schneider JA, Ardestani BM, Young LE (2020). Individual and social network structure characteristics associated with peer change agent engagement and impact in a PrEP intervention. AIDS Behav.

[57] Felsher M, Koku E, Bellamy SL, Mulawa MI, Roth AM (2021). Predictors of willingness to diffuse PrEP information within ego-centric networks of women who inject drugs. AIDS Behav.

[58] Crawford ND, Myers S, Young H, Klepser D, Tung E (2021). The role of pharmacies in the HIV prevention and care continuums: a systematic review. AIDS Behav.

[59] Amster R (2003). Patterns of exclusion: sanitizing space, criminalizing homelessness. Social Justice.

[60] Robinson T (2017). No right to rest: police enforcement patterns and quality of life consequences of the criminalization of homelessness. Urban Affairs Review.

[61] Barry-Jester AM (2020). Sweeps of homeless camps in California aggravate key health issues. NPR.

[62] Darrah-Okike J, Soakai S, Nakaoka S, Dunson-Strane T, Umemoto K (2018). “It Was Like I Lost Everything”: the harmful impacts of homeless-targeted policies. Housing Policy Debate.

